# Endometriosis does not confer improved prognosis in ovarian clear cell carcinoma: a retrospective study at a single institute

**DOI:** 10.1186/s13048-018-0425-9

**Published:** 2018-06-26

**Authors:** Ting Zhao, Yu Shao, Yan Liu, Xiao Wang, Luyao Guan, Yuan Lu

**Affiliations:** Department of Gynecology, Obstetrics and Gynecology Hospital of Fudan University, 419 Fangxie Road, Shanghai, 200011 China

**Keywords:** Clear cell carcinoma, Chemosensitivity, Endometriosis, Ovarian cancer, Survival

## Abstract

**Background:**

Considered as the precursor lesion of a subset of ovarian clear cell carcinoma (OCCC), the prognostic role of endometriosis in OCCC patients remains controversial. This study aimed to investigate the prognostic role of coexisting endometriosis in the survival of patients with OCCC, and also sought to identify other prognostic factors.

**Results:**

A total of 125 patients were diagnosed with OCCC during the study period. Of these, 55 (44.0%) patients had coexisting endometriosis. Patients with endometriosis were younger (*p* = 0.030), had smaller tumor diameter (*p* = 0.005) and lower preoperative CA125 levels (*p* = 0.005). More patients with endometriosis had International Federation of Gynecology and Obstetrics (FIGO) stage I disease (83.6% vs. 51.4%, *p* = 0.000) and exhibited sensitivity to platinum-based regimen (89.6% vs. 66.7%, *p* = 0.003). Univariate and multivariate analysis revealed that coexisting endometriosis was not a predictor of 5-year overall survival (OS) or progression-free survival (PFS) of OCCC patients. For OS, chemosensitivity was the only useful prognostic factor (Hazards ratio (HR) 109.33, 95% Confidence Interval (CI) 23.46–511.51; *p* = 0.000). For PFS, the useful prognostic factors were ascites (HR 2.78, 95% CI 1.21–6.47; *p* = 0.016), FIGO stage (HR 1.61, 95% CI 1.04–2.49; *p* = 0.033), and chemosensitivity (HR 101.60, 95% CI 29.45–350.49; *p* = 0.000). Moreover, higher FIGO stage was the only risk factor for resistance to platinum-based chemotherapy (Exp (B) = 0.292, 95% CI 0.123–0.693; *p* = 0.005).

**Conclusions:**

In this study, coexisting endometriosis was not a prognostic factor for the survival of OCCC patients. The most important predictor of both 5-year OS and PFS was chemosensitivity to platinum-based regimen, which decreased significantly with increase in FIGO stage.

## Background

Ovarian clear cell carcinoma (OCCC) is the second most common histological subtype of epithelial ovarian carcinoma (EOC) after high-grade serous carcinoma (HGSC) and accounts for > 10% of EOC [1, 2]. OCCC typically presents as a large unilateral pelvic mass and is frequently diagnosed at an early stage [3]. Unlike HGSC, this subtype of EOC is typically insensitive to conventional platinum-based chemotherapy [4]. As a result, it has a poorer prognosis as compared to that of HGSC of comparable stage [5]. In the absence of alternative chemotherapy regimens, treatment of patients with OCCC represents a clinical challenge [4].

Endometriosis is a common gynecological condition that affects 5–20% of premenopausal women [6]. It is a benign condition that exhibits some characteristics of malignant disease such as tissue invasion and distant spread [7]. In 1925, Sampon first described a case of endometriosis that transformed to ovarian carcinoma [8]. Subsequently, a consistent body of evidence has addressed the relationship between endometriosis and certain EOC subtypes. A pooled analysis published in *Lancet* showed that self-reported endometriosis was associated with a significantly increased risk of OCCC [Odds ratio (OR) 3.05, 95% Confidence Interval (CI) 2.43–3.84)], ovarian endometrioid carcinoma (OEC) (OR 2.04, 95% CI 1.67–2.48), and low-grade serous carcinoma (OR 2.11, 95% CI 1.39–3.20) [9]. Histopathology studies have also provided compelling evidence that endometriosis is a precursor lesion for OCCC and OEC [3].

It was suggested that OCCC is distinct disease entity from other endometriosis-associated ovarian tumors (EAOCs) with a distinct gene expression profile. As reported by a number of researchers, hepatocyte nuclear factor 1β (HNF-1β) was exclusively expressed in almost all OCCC cases, but not in other EOCs including OEC [10–12]. Positive expression of HNF-1β was detected in 61.1% of ovarian endometriod cysts [13]. It was subsequently extrapolated that OCCC arises from HNF-1β positive epithelial cells while OEC arises from HNF-1β negative epithelial cells of endometriosis [13].

Theoretically, only a subset of OCCC is derived from endometriosis [9]. Yet controversy still remains regarding the prognostic role of endometriosis in OCCC patients [14]. So far, most of the studies that have investigated the prognostic role of endometriosis in the context of EAOC have included multiple histological types, while OCCC only consisted a small subgroup of the subjects [14]. Some studies precluded other subtypes but included OCCC mixed with other histological types such as serous carcinoma [15]. Owing to heterogeneity with respect to histological subtypes of EAOCs, the results of these studies should be interpreted with caution. Only a few studies have focused exclusively on pure OCCC. Of these, 3 studies reported series of 47 [16], 55 [17] and 84 [3] patients, respectively. The sample size in these studies was limited and the last study spanned over a period of 27 years, during which time considerable changes in adjuvant therapies took place. The other studies also did not reach a consensus about the prognostic role of endometriosis [2, 18, 19]. The purpose of the present study was to investigate whether concomitant endometriosis affects the survival of patients with pure OCCC and to identify other prognostic factors in these patients.

## Methods

This was a retrospective study approved by the ethics committee of the OB/GYN Hospital of Fudan University. The inclusion criteria were: [1] patients who underwent primary surgery in the hospital between January 1995 and December 2014; [2] histological diagnosis of pure OCCC. The exclusion criteria were: [1] patients with mixed histological subtypes such as OCCC with high-grade serous carcinoma or endometrioid carcinoma; [2] patients with concurrent genital or extra-genital primary malignancy.

**Fig. 1:**
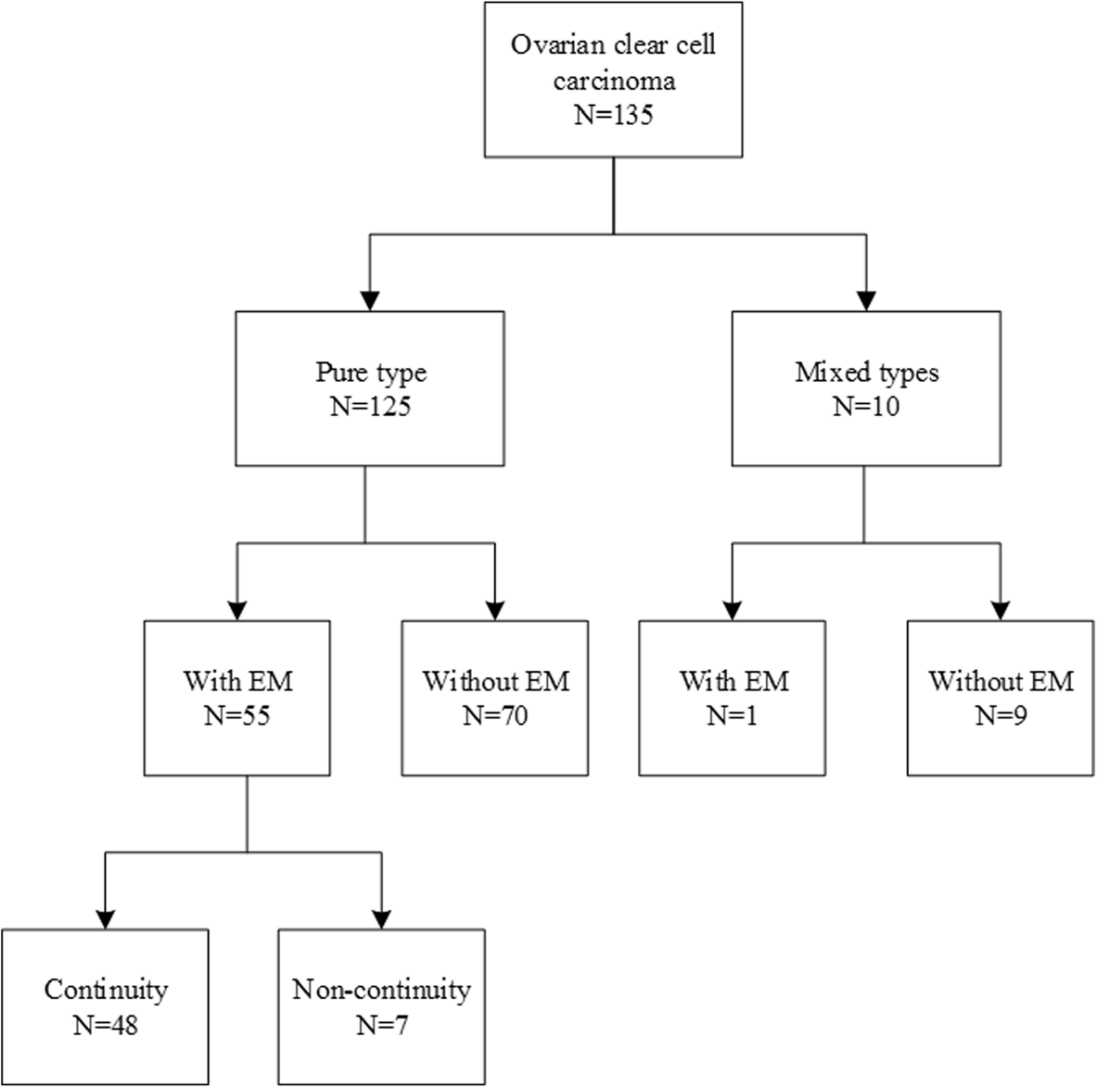
An overview of the subject of this study. A total of 135 cases were diagnosed with ovarian clear cell carcinoma in the study period. Finally 125 cases were included in the analysis and 10 cases were excluded for mixed subtypes. There were 55 cases with endometriosis while 70 cases without. Of the 55 patients, continuity of clear cell carcinoma from endometriosis was detected in 48 cases. EM: endometriosis. Continuity: continuity of clear cell carcinoma from endometriosis.

A total of 135 patients were diagnosed with ovarian clear cell carcinoma in the study period. All the patients received primary surgery in our institute and none of them had concurrent  primary malignancies of other organs. Of these, there were 10 patients diagnosed with mixed types according to the pathological reports (Fig. 1). Finally 125 patients were included in this study and were divided into two groups based on the presence or absence of endometriosis. OCCC with endometriosis was defined as endometriosis involving the same or the contralateral ovary or the pelvic peritoneum of the same patient. All the histological slides were independently reviewed by two pathologists.

A comprehensive review of the medical records was performed. Data pertaining to the following variables were obtained: age at diagnosis; personal medical history; reproductive history; preoperative level of CA125; ultrasonography findings; surgery details; adjuvant chemotherapy; date of disease progression or death; and status of the patient at the most recent follow-up. Comprehensive surgical staging was defined according to International Federation of Gynecology and Obstetrics (FIGO) guidelines (version 2015) for ovarian cancer. Satisfactory debulking surgery was defined as residual lesion ≤1 cm. Platinum-sensitivity was defined as relapse occurring ≥6 months after the completion of last regimen or lack of recurrence. Platinum-resistance was defined as relapse occurring within 6 months of the completion of last regimen.

The expressions of tumor suppressor gene protein p53, cell proliferation index Ki-67, estrogen receptor (ER) and progesterone receptor (PR) were evaluated using standard immunoperoxidase technique. The immunoreactivity was determined by counting the positively stained nuclei in at least 100 cells of the tumor tissue samples. Ki-67 immunoreactivity was expressed as a percentage. ER, PR, and p53 expressions were scored semiquantitatively as 0 (< 5% positive cells in 10 × HPF), 1 (5–25%), 2 (25–50%), 3 (51–75%), or 4 (75–100%).

Statistical analysis was performed using SPSS software (version 16.0, Chicago, IL, USA). All data are expressed as mean ± standard deviation (SD). Between-group differences with respect to continuous variables were assessed by *t*-test or Mann-Whitney test, as appropriate. The Pearson Chi-square test or Fisher’s exact test was used to assess differences with respect to categorical variables. Spearman’s correlation analysis was used to assess the correlation between variables. Variables with *p <* 0.05 were included in the logistic regression model. Overall survival (OS) and progression-free survival (PFS) was calculated from the date of primary surgery to death and recurrence, respectively, or the last disease-free visit. Survival analysis was performed using Kaplan-Meier model. Variables associated with *p* values < 0.1 in univariate analyses were included in the Cox regression model to account for the confounding factors. All *p* values reported are two-tailed and a *p <* 0.05 was considered significant.

## Results

### Characteristics of the study population

**Fig. 2:**
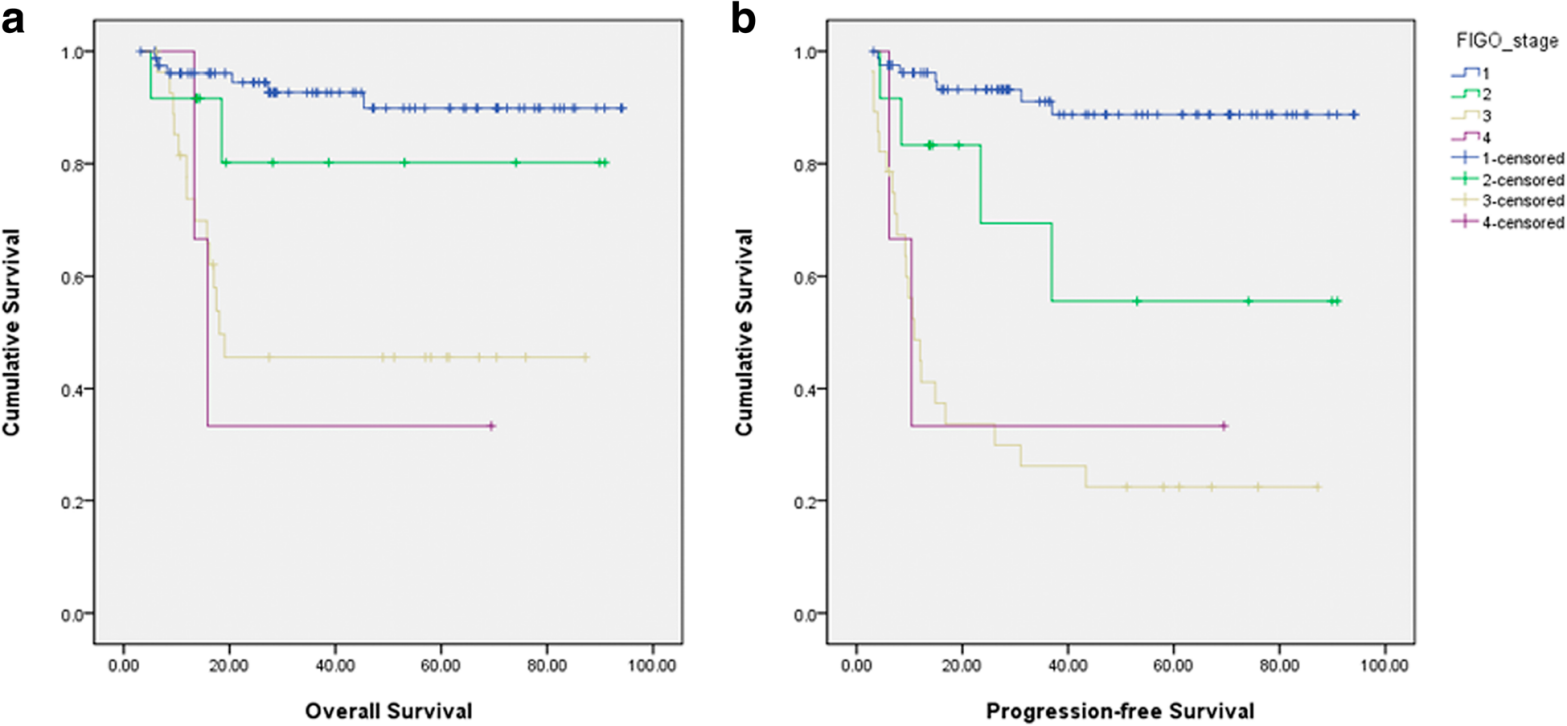
Survival curves for overall survival (OS) and progression-free survival (PFS) of patients in this study. **a** OS curves for patients of different stages; **b** PFS curves for patients of different stages.

A total of 125 patients treated during the 19-year study period qualified the inclusion and exclusion criteria. Mean age at the time of diagnosis was 51.6 ± 7.8 years (range, 31–75). Mean tumor diameter was 11.3 ± 4.6 cm (range, 2.3–25.2) (Table 1). Over a median follow-up period of 28.9 months (range, 3.2–93.9 months), 5 patients (4.0%) were lost to follow-up. Twenty-four disease-specific deaths were observed. The 5-year OS and PFS for the entire study cohort was 78.7 and 74.5%, respectively. In total, 82 (65.6%), 12 (9.6%), 28 (22.4%), and 3 (2.4%) patients were diagnosed with FIGO stage I, II, III, and IV, respectively. Most patients with FIGO stage I and IIA disease received comprehensive staging surgery including total hysterectomy, salpingo-oophorectomy, pelvic lymphadnectomy, and omentum resection; however, 3, 2, and 1 patient with FIGO stage I disease only received hysterectomy and salpingo-oophorectomy, salpingo-oophorectomy, and ovarian cystectomy, respectively. All patients with FIGO stage IIB disease or higher received debulking surgery. The 5-year OS of patients with FIGO stage I, II, III and IV was 89.9, 80.2, 45.5, and 33.3%, respectively (*p* = 0.000). The corresponding 5-year PFS was 88.8, 55.6, 22.4, and 33.3%, respectively (*p* = 0.000) (Fig. 2). There were 122 patients who received platinum-based chemotherapy regimen after surgery while 3 patients with FIGO stage I disease did not receive chemotherapy. Of these, 119 patients received TP or TC regimen (paclitaxel taxol 135 mg/m^2^ and carboplatin AUC (area under the curve) = 5, or cisplatin 75 mg/m^2^); 1 patient with FIGO stage I received PAC regimen (cisplatin, doxorubicin, and cyclophosphamide); 1 patient with FIGO stage IIIC disease received PEFC regimen (carboplatin, etoposide, fluorouracil, and cyclophosphamide); and 1 patient with FIGO stage IV disease received TVP regimen (paclitaxel taxol, etoposide, and cisplatin). Most (96.2%, 76/79) patients with FIGO stage I disease received ≥4 courses and most (95.3%, 41/43) patients with FIGO stage II disease or higher received ≥6 courses of regimen. For 11 out of the 122 patients, the status pertaining to chemosensitivity could not be verified due to the following reasons: [1] loss of follow-up for 5 patients (4 patients with FIGO stage I and 1 patient with FIGO stage III); [2] Until the cut-off date for the present study the follow-up time was not long enough to assess the status of 6 patients (all with FIGO stage I disease). Among the other patients, 76.6% (85/111) patients were sensitive to platinum-based chemotherapy. Specifically, 91.4% (74/81) of patients with FIGO stage I or II disease exhibited chemosensitivity; for patients with advanced disease (FIGO stage III and IV), the response rate was 36.7% (11/30).

**Table 1:** Clinicopathological features of patients with ovarian clear cell carcinoma

Characteristics	All (*N* = 125)	NEM (*N* = 70)	EM (*N* = 55)	*p* value
Age (years)	51.6 ± 7.8	52.9 ± 8.5	49.9 ± 6.4	0.030
Parity	0.9 ± 0.5	1.0 ± 0.5	0.8 ± 0.5	0.040
Nulliparous (%)	20 (16.1%) (*n* = 124)	8 (11.6%) (*n* = 69)	12 (21.8%) (*n* = 55)	0.145
Tumor size (cm)	11.3 ± 4.6 (*n* = 118)	12.3 ± 5.0 (*n* = 66)	9.9 ± 3.7 (*n* = 52)	0.005
Serum CA 125 (U/mL)	295.0 ± 934.7	433.5 ± 1188.2	94.7 ± 198.4	0.005
Normal (%)	40 (34.8%)	20 (29.4%)	20 (42.6%)	0.167
Elevated (%)	75 (65.2%) (*n* = 115)	48 (70.6%) (*n* = 68)	27 (57.4%) (*n* = 47)	
Laterality (%)				0.045
Left	61 (48.8%)	32 (45.7%)	29 (52.7%)	
Right	52 (41.6%)	28 (40.0%)	24 (43.6%)	
Both	12 (9.6%)	10 (14.3%)	2 (3.6%)	
FIGO stage (%)				0.000
I	82 (65.6%)	36 (51.4%)	46 (83.6%)	
II	12(9.6%)	7 (10.0%)	5 (9.1%)	
III	28 (22.4%)	24 (34.3%)	4 (7.3%)	
IV	3 (2.4%)	3 (4.3%)	0 (0%)	
Surgical method (%)				0.070
Laparoscopy	21 (16.8%)	8 (11.4%)	13 (23.6%)	
Laparotomy	104 (83.2%)	62 (88.6%)	42 (76.4%)	
Comprehensive staging (%)				0.829
Yes	114 (91.2%)	63 (90.0%)	51 (92.7%)	
No	11 (8.8%)	7 (10.0%)	4 (7.3%)	
Ascites (%)	48 (38.4%)	37 (52.9%)	11 (20%)	0.000
Ascites positivity (%)	20 (41.7%)	18 (48.6%)	2 (18.2%)	0.147
Lymph nodes status (%)				0.031
Positive	12 (10.6%)	10 (16.9%)	2 (1.8%)	
Negative	101 (89.4%) (*n* = 113)	49 (83.1%) (*n* = 59)	52 (96.3%) (*n* = 54)	
Residual lesion (%)	120 (96.0%)	66 (94.3%)	54 (98.2%)	0.520
0	113 (90.4%)	60 (85.7%)	53 (96.4%)	
≤1 cm	7 (5.6%)	6 (8.6%)	1 (1.8%)	
> 1 cm	5 (4.0%)	4 (5.7%)	1 (1.8%)	
Cycles of chemotherapy	6.3 ± 1.8	6.4 ± 1.9	6.1 ± 1.6	0.382
Chemosensitivity (%)				0.003
Sensitive	85 (76.6%)	42 (66.7%)	43 (89.6%)	
Resistant	26 (23.4%) (*n* = 111)	21 (33.3%) (*n* = 63)	5 (10.4%) (*n* = 48)	

*EM* endometriosis, *NEM* without endometriosis

Of the study population, 55 (44.0%) patients with endometriosis were assigned to EM group; 70 patients (56.0%) with no evidence of endometriosis were assigned to NEM group.

### Comparison of clinical and pathological features between the two groups

The clinical parameters of the two groups are shown in Table 1. Compared to NEM group, patients in the EM group were younger (49.9 ± 6.4 vs.52.9 ± 8.5 years, *p* = 0.030), gave fewer births (0.8 ± 0.5 vs.1.0 ± 0.5, *p* = 0.040), and had smaller tumor diameter (9.9 ± 3.7 vs. 12.3 ± 5.0 cm, *p* = 0.005), and lower preoperative CA125 levels (94.7 ± 198.4 vs. 433.5 ± 1188.2 U/ml, *p* = 0.005). More patients in the EM group had FIGO stage I disease (83.6% vs. 51.4%, *p* = 0.000). On the contrary, a lower proportion of patients in the EM group showed bilateral involvement (3.6% vs. 14.3%, *p* = 0.045), lymph node involvement (1.8% vs. 16.9%, *p* = 0.031), or ascites (20.0% vs. 52.9%, *p* = 0.000). A significantly greater proportion of patients in the EM group were sensitive to platinum-based regimen (89.6% vs. 66.7%, *p* = 0.003).

No significant between-group difference was observed with respect to the proportion of patients who underwent laparotomy, the ratio of patients who received satisfactory debulking surgery and chemotherapy cycles. Immunoreactivity of ER, PR, p53, and Ki-67 was also comparable between the two groups (Table 2).

**Table 2:** Immunohistochemical staining intensity of ER, PR, p53, and Ki-67 in ovarian clear cell carcinoma patients

Index	NEM	EM	*p* value^a^
ER	0.50 ± 1.00	0.56 ± 0.91	0.478
PR	0.21 ± 0.51	0.12 ± 0.44	0.207
p53	0.93 ± 0.97	0.77 ± 0.61	0.769
Ki-67	32.54 ± 24.15	32.35 ± 19.30	0.662

*EM* endometriosis, *NEM* without endometriosis

^a^Mann-Whitney test

### Comparison of survival outcomes between the two groups

**Fig. 3:**
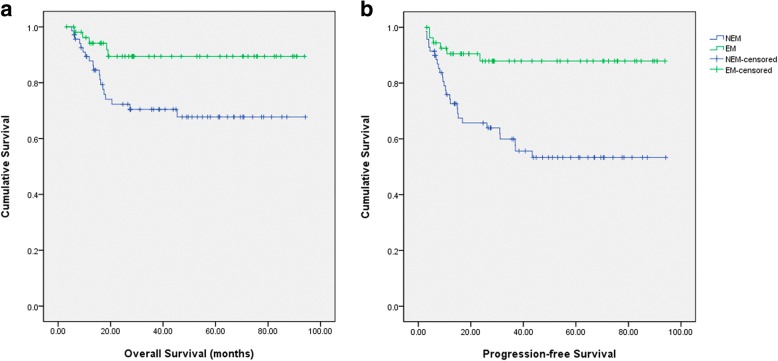
Survival curves for overall survival (OS) and progression-free survival (PFS) in the two groups. **a** OS curves for two groups. **b** PFS curve for two groups. NEM: patients without endometriosis; EM: endometriosis.

As estimated from Kaplan-Meier survival curves, both the 5-year OS and PFS in the EM group were better than that in the NEM group (OS: 89.4% vs. 67.7%, *p* = 0.013) (PFS: 87.9% vs. 53.3%, *p* = 0.001) (Fig. 3).

As some features such as age, FIGO stage, and preoperative CA125 level were different between the two groups, Cox regression model was used to account for the influence of these factors. No significant between-group difference with respect to survival was observed after controlling for these confounding factors (*p* > 0.05).

Of the 55 patients from EM group, there were 48 cases had endometriosis in direct continuity from the OCCC lesion, and seven cases not (Fig. 1). We also compared the survival outcomes between them. They were comparable in 5-year OS (92.7% vs. 71.4%, *p* = 0.083) but significant difference of 5-year PFS (93.4% vs. 53.6%, *p* = 0.004) was observed. However, after controlling for confounding factors such as FIGO stage, no significant difference with respect to PFS was observed in Cox regression model (*p* = 0.607).

### Predictors of survival in univariate and multivariate survival analysis

We performed the *log*-rank test using *p* < 0.1 as a criteria to identify predictors of survival. The potential prognostic factors for PFS were: presence of endometriosis, preoperative CA125 level, bilateralism, tumor diameter, size of residual lesion, ascites, lymph node positivity, FIGO stage, number of chemotherapy cycles, and chemosensitivity. The potential prognostic factors for OS were: presence of endometriosis, bilateralism, comprehensive staging, size of residual lesion, ascites, lymph node positivity, FIGO stage, and chemosensitivity.

After recalculation in Cox regression model, the useful prognostic factors for PFS were ascites (Hazards ratio (HR) 2.78, 95% CI1.21–6.47; *p* = 0.016), FIGO stage (HR 1.61, 95% CI1.04–2.49; *p* = 0.033), and chemosensitivity (HR 101.60, 95% CI29.45–350.49; *p* = 0.000). For OS, chemosensitivity was the only useful prognostic factor (HR 109.33, 95% CI23.46–511.51; *p* = 0.000) (Table 3).

**Table 3:** Prognostic factors for progression-free survival (PFS) and overall survival (OS) of ovarian clear cell carcinoma patients

Variables	5 year-PFS %	*p* value^a^	*p* value^b^	HR (95%CI)	5 year-OS %	*p* value^a^	*p* value^b^	HR (95%CI)
Endometriosis		0.001	0.295			0.013	0.677	
With	87.9				89.4			
Without	53.3				67.7			
Age (years)		0.628				0.550		
< 60	72.0				79.6			
≥60	47.8				69.6			
CA-125 (U/mL)		0.001	0.620			0.142		
< 200	76.6				80.0			
≥200	38.4				64.4			
Parity		0.342				0.382		
0	70.8				89.5			
≥1	53.6				75.9			
Laterality		0.034	0.093			0.028	0.360	
Unilateral	70.1				79.3			
Bilateral	46.7				56.2			
Tumor diameter		0.084	0.662			0.236		
< 10	77.7				83.4			
≥10	59.8				70.0			
Comprehensive staging		0.348				0.070	0.441	
Yes	68.6				79.0			
No	58.9				56.2			
Residual lesion		0.000	0.126			0.001	0.731	
Yes	72.8				82.6			
No	25.0				33.3			
Ascites		0.000	0.016			0.001	0.052	
Yes	45.9			2.78 (1.21–6.47)	60.8			
No	82.0				87.6			
Lymph nodes status		0.000	0.324			0.012	0.395	
Positive	33.3				58.3			
Negative	76.0				85.6			
FIGO stage		0.000	0.033	1.61 (1.04–2.49)		0.000	0.874	
I	88.8				89.9			
II	55.6				80.2			
III	22.4				45.5			
IV	33.3				33.3			
Chemotherapy cycles		0.050	0.290			0.421		
≥6	72.5				78.4			
> 6	49.9				69.5			
Chemosensitivity		0.000	0.000	101.60 (29.45–350.49)		0.000	0.000	109.55
Sensitive	87.3				96.5			(23.46–511.51)
Resistant	0				9.2			

^a^log-rank test; ^b^ Cox-proportional hazards model

### Factors related to chemosensitivity in the logistic regression model

As chemosensitivity was the most important prognostic factor for both OS and PFS of patients with OCCC, further analysis was performed to identify covariates of chemosensitivity. Spearman’s correlation analysis showed that preoperative CA125 level, ascites, size of residual lesion, lymph node positivity, FIGO stage, and presence of endometriosis were associated with chemosensitivity (*p* < 0.05). However, after entering into logistic regression equation, only FIGO stage was significantly associated with chemosensitivity (Exp (B) = 0.292, 95% CI0.123–0.693; *p* = 0.005) (Table 4).

**Table 4:** Risk factors for chemosensitivity to platinum-based regimen of ovarian clear cell carcinoma patients

Variables	*p* value^a^	Correlation Coefficent	*p* value^b^	Exp (B) (95% CI)
Age	0.995			
Tumor diameter	0.396			
CA125 value	0.028	−0.216	0.887	
Number of live birth	0.448			
Ascites	0.004	−0.267	0.673	
Residual lesion	0.000	−0.348	0.467	
Lymph nodes positivity	0.002	−0.306	0.858	
FIGO stage	0.000	−0.546	0.005	0.292 (0.123–0.693)
Bilaterality	0.060			
Endometriosis	0.003	0.279	0.671	
ER	0.579			
PR	0.191			
p53	0.990			
Ki67	0.681			

^a^Spearman’s correlation test; ^b^Logistic regression model

## Discussion

Pathological evidence of the synchronous presence of endometriosis and OCCC has been consistently reported [20]. A review of 15 published reports concluded that 39.2% of patients with OCCC had coexisting endometriosis [21]. In the present study, this percentage was 44.0% (55/125), which is comparable to the reported figure. The histological characteristics of OCCC include large cuboidal, hobnailed or flattened epithelial cells containing abundant clear cytoplasm lining the tubules and cysts, and exhibiting a solid/tubular or glandular growth pattern [22]. The hobnail cells present a very strong morphological resemblance to endometrial Arias-Stella cells [23].

The mechanism by which endometriosis develops into OCCC remains largely unknown. It was suggested that OCCC develops in a step-wise fashion from endometriosis through metaplastic, hyperplastic, and atypical endometriosis [24]. Atypical endometriosis is characterized by epithelial cells showing nuclear enlargement, crowding, slight hyperchromasia, and possible chromocentres/nucleoli architectural abnormalities [25]. It was reported that OCCC-associated endometriosis already harbors aberrant gene expression, such as altered expressions of *eEF1A2, PTCH2, PPP1R14B*, and *XRCC5*, which may not be found in endometriosis tissue in the absence of cancer [26]. Shared gene alterations such as *PTEN*, *PIK3CA* or *ARID1A* mutation were documented between ovarian cancers and adjacent normal-appearance endometriosis, which suggests that these gene mutations represent early events in the carcinogenic pathway before the appearance of the atypical precancerous lesions [27–29]. Moreover, HNF-1β expression is significantly increased and ER expression is significantly down-regulated in primary OCCC lesions as compared to that in matched endometriosis, which suggests that the changes in these proteins were relatively late carcinogenic events [26]. The product of *H3K27me3* gene sets and one of its target proteins, WT1, were found enriched in neighboring endometriosis, but silenced in OCCC lesion, which suggests that epigenetic reprogramming transformed the endometriotic cells to a pluripotent stage as OCCC [26].

Compared to NEM group, patients in the EM group presented favorable characteristics such as younger age and earlier stage, which is consistent with previously reported results [7, 30]. This is likely attributable to the typical symptoms of endometriosis which may facilitate earlier attendance to the clinics [7, 30]. Moreover, in advanced stage malignancies the tumor might have masked the deriving tissue of origin; this may have contributed to the lower frequency of detection of endometriosis [31].

Some studies have suggested that OCCC patients with concomitant endometriosis have a better prognosis than that of patients without endometriosis [31]. However, other studies have found no difference in survival after adjusting for stage and age [7, 32, 33]. A meta-analysis of evidence from 10 cohort studies also concluded that endometriosis is not a prognostic factor for the survival of OCCC patients [34]. A study conducted in Korea found that only in FIGO stage I patients was endometriosis an independent prognostic factor [19]. However, studies conducted in China found no prognostic role of endometriosis in OCCC patients [2], even when the tumor was confined to FIGO stage I [18]. In the present study, either, endometriosis was not found to be a prognostic factor in OCCC patients. These results collectively suggest that endometriosis may not affect the progression after the onset of ovarian cancer [7]. Moreover, there was no difference with respect to key molecular characteristics such as the expressions of p53, Ki-67, ER and PR between the two groups, which also suggests homogeneity across the two groups.

In the present study, the most important prognostic factor for survival was the sensitivity to platinum-based chemotherapy. With a HR of 109.55 (95% CI 23.46–511.51) for 5-year OS and 101.60 (95% CI 29.45–350.49) for PFS, it is reasonable to conclude that chemosensitivity to platinum-based chemotherapy was a key determinant of the survival of OCCC patients. For 5-year OS, chemosensitivity was the only prognostic factor while FIGO stage was not. However, FIGO stage was the only risk factor for chemosensitivity in the logistic regression equation. Specifically, the higher the FIGO stage, the more the resistance to platinum-based chemotherapy. Patients affected by localized (FIGO stage I and II) OCCC exhibited a response rate of 91.4% to platinum-based chemotherapy while their counterparts with advanced disease (FIGO stage III and IV) exhibited a response rate of only 36.7%. The prognosis of patients with FIGO stage I and II OCCC was reported to be comparable to that of serous adenocarcinoma, while the prognosis of patients with FIGO stage III and IV disease was much poorer compared to that of serous adenocarcinoma [4, 35, 36]. The reported median survival time for patients with advanced disease was only 12.7–23.0 months [23, 37, 38]. It is generally recognized that the poor survival of patients with advanced stage disease is largely due to the insensitivity to conventional platinum-based chemotherapy [39]. The mechanism of resistance includes decreased drug efflux, increased drug inactivation, and increased DNA repair activity [39]. These results indicate that platinum-based chemotherapy may not be the optimum treatment regimen for patients with advanced OCCC. However, the National Comprehensive Cancer Network (NCCN) guidelines (version 2016) still recommend treatment of OCCC with platinum-based chemotherapy. New molecular targets have been identified and experimented with in the past decades such as mitogen-activated protein kinase kinase (MAPK), phosphatidylinositol 3’-kinase (PI3K) signaling pathway [40], and epidermal growth-factor receptor (EGFR) [41]. However, no definite consensus has been reached about the efficacy of the above strategies.

## Conclusions

Although this work has limitations inherent to retrospective studies, to the best of our knowledge, this is the largest study to compare patients diagnosed with OCCC with and without concomitant endometriosis. The two groups in this study were heterogeneous in many aspects such as age and FIGO stage. However, the prognosis was not different after controlling for confounding factors. The most important prognostic factor for the survival of OCCC patients was the chemosensitivity to platinum-based chemotherapy, which showed an inverse correlation with FIGO stage. This study calls for further research to unravel the mechanism of development of chemoresistance and underlines the need to develop novel therapeutic strategies for patients with advanced stage OCCC.

## Abbreviations

AUC: Area under the curve
CI: Confidence interval
EAOCs: Endometriosis-associated ovarian tumors
EGFR: Epidermal growth-factor receptor
EOC: Epithelial ovarian carcinoma
ER: Estrogen receptor
FIGO: International Federation of Gynecology and Obstetrics
HGSC: High-grade serous carcinoma
HNF-1β: Hepatocyte nuclear factor 1β
HR: Hazards ratio
MAPK: Mitogen-activated protein kinase kinase
NCCN: National Comprehensive Cancer Network
OCCC: Ovarian clear cell carcinoma
OEC: Ovarian endometrioid carcinoma
OS: Overall survival
OR: Odds ratio
PFS: Progression-free survival
PI3K: Phosphatidylinositol 3’-kinase
PR: Progesterone receptor
SD: Standard deviation

## References

[1] Kobel M, Kalloger SE, Huntsman DG, Santos JL, Swenerton KD, Seidman JD (2010). Differences in tumor type in low-stage versus high-stage ovarian carcinomas. Int J Gynecol Pathol.

[2] Ye S, Yang J, You Y, Cao D, Bai H, Lang J (2014). Comparative study of ovarian clear cell carcinoma with and without endometriosis in People's Republic of China. Fertil Steril.

[3] Orezzoli JP, Russell AH, Oliva E, Del Carmen MG, Eichhorn J, Fuller AF (2008). Prognostic implication of endometriosis in clear cell carcinoma of the ovary. Gynecol Oncol.

[4] Sugiyama T, Kamura T, Kigawa J, Terakawa N, Kikuchi Y, Kita T (2000). Clinical characteristics of clear cell carcinoma of the ovary: a distinct histologic type with poor prognosis and resistance to platinum-based chemotherapy. Cancer.

[5] Chan JK, Teoh D, JM H, JY Shin KO, Kapp DS (2008). Do clear cell ovarian carcinomas have poorer prognosis compared to other epithelial cell types? A study of 1411 clear cell ovarian cancers. Gynecol Oncol.

[6] Bulun SE (2009). Endometriosis. N Engl J Med.

[7] Kim HS, Kim TH, Chung HH, Song YS (2014). Risk and prognosis of ovarian cancer in women with endometriosis: a meta-analysis. Br J Cancer.

[8] Sampson J (1925). Endometrial carcinoma of the ovary arising in endometrial tissue in that organ. Arch Surg.

[9] Pearce CL, Templeman C, MA Rossing A, Lee AM (2012). Near, PM Webb et al. association between endometriosis and risk of histological subtypes of ovarian cancer: a pooled analysis of case-control studies. Lancet Oncol.

[10] Tsuchiya A, Sakamoto M, Yasuda J, Chuma M, Ohta T, Ohki M (2003). Expression profiling in ovarian clear cell carcinoma: identification of hepatocyte nuclear factor-1 beta as a molecular marker and a possible molecular target for therapy of ovarian clear cell carcinoma. Am J Pathol.

[11] Kato N, Sasou S, Motoyama T (2006). Expression of hepatocyte nuclear factor-1beta (HNF-1beta) in clear cell tumors and endometriosis of the ovary. Mod Pathol.

[12] Yamamoto S, Tsuda H, Aida S, Shimazaki H, Tamai S, Matsubara O (2007). Immunohistochemical detection of hepatocyte nuclear factor 1beta in ovarian and endometrial clear-cell adenocarcinomas and nonneoplastic endometrium. Hum Pathol.

[13] Kajihara H, Yamada Y, Shigetomi H, Higashiura Y, Kobayashi H (2012). The dichotomy in the histogenesis of endometriosis-associated ovarian cancer: clear cell-type versus endometrioid-type adenocarcinoma. Int J Gynecol Pathol.

[14] Scarfone G, Bergamini A, Noli S, Villa A, Cipriani S, Taccagni G (2014). Characteristics of clear cell ovarian cancer arising from endometriosis: a two center cohort study. Gynecol Oncol.

[15] Schnack TH, Hogdall E, Thomsen LN, Hogdall C (2017). Demographic, clinical, and prognostic factors of ovarian clear cell adenocarcinomas according to endometriosis status. Int J Gynecol Cancer.

[16] Komiyama S, Aoki D, Tominaga E, Susumu N, Udagawa Y, Nozawa S (1999). Prognosis of Japanese patients with ovarian clear cell carcinoma associated with pelvic endometriosis: clinicopathologic evaluation. Gynecol Oncol.

[17] Noli S, Cipriani S, Scarfone G, Villa A, Grossi E, Monti E (2013). Long term survival of ovarian endometriosis associated clear cell and endometrioid ovarian cancers. Int J Gynecol Cancer.

[18] Bai H, Cao D, Yuan F, Sha G, Yang J, Chen J (2016). Prognostic value of endometriosis in patients with stage I ovarian clear cell carcinoma: experiences at three academic institutions. Gynecol Oncol.

[19] Park JY, Kim DY, Suh DS, Kim JH, Kim YM, Kim YT (2018). Significance of ovarian endometriosis on the prognosis of ovarian clear cell carcinoma. Int J Gynecol Cancer.

[20] Vigano P, Somigliana E, Parazzini F, Vercellini P (2007). Bias versus causality: interpreting recent evidence of association between endometriosis and ovarian cancer. Fertil Steril.

[21] Yoshikawa H, Jimbo H, Okada S, Matsumoto K, Onda T, Yasugi T (2000). Prevalence of endometriosis in ovarian cancer. Gynecol Obstet Investig.

[22] Scully RE (1970). Recent progress in ovarian cancer. Hum Pathol.

[23] Aure JC, Hoeg K, Kolstad P (1971). Clinical and histologic studies of ovarian carcinoma. Long-term follow-up of 990 cases. Obstet Gynecol.

[24] Prefumo F, Todeschini F, Fulcheri E, Venturini PL (2002). Epithelial abnormalities in cystic ovarian endometriosis. Gynecol Oncol.

[25] King CM, Barbara C, Prentice A, Brenton JD, Charnock-Jones DS (2016). Models of endometriosis and their utility in studying progression to ovarian clear cell carcinoma. J Pathol.

[26] Worley MJ, Liu S, Hua Y, Kwok JS, Samuel A, Hou L (2015). Molecular changes in endometriosis-associated ovarian clear cell carcinoma. Eur J Cancer.

[27] Sato N, Tsunoda H, Nishida M, Morishita Y, Takimoto Y, Kubo T (2000). Loss of heterozygosity on 10q23.3 and mutation of the tumor suppressor gene PTEN in benign endometrial cyst of the ovary: possible sequence progression from benign endometrial cyst to endometrioid carcinoma and clear cell carcinoma of the ovary. Cancer Res.

[28] Wiegand KC, Shah SP, Al-Agha OM, Zhao Y, Tse K, Zeng T (2010). ARID1A mutations in endometriosis-associated ovarian carcinomas. N Engl J Med.

[29] Yamamoto S, Tsuda H, Takano M, Tamai S, Matsubara O (2012). Loss of ARID1A protein expression occurs as an early event in ovarian clear-cell carcinoma development and frequently coexists with PIK3CA mutations. Mod Pathol.

[30] Wang S, Qiu L, Lang JH, Shen K, Yang JX, Huang HF (2013). Clinical analysis of ovarian epithelial carcinoma with coexisting pelvic endometriosis. Am J Obstet Gynecol.

[31] Somigliana E, Vigano P, Parazzini F, Stoppelli S, Giambattista E, Vercellini P (2006). Association between endometriosis and cancer: a comprehensive review and a critical analysis of clinical and epidemiological evidence. Gynecol Oncol.

[32] Erzen M, Rakar S, Klancnik B, Syrjanen K (2001). Endometriosis-associated ovarian carcinoma (EAOC): an entity distinct from other ovarian carcinomas as suggested by a nested case-control study. Gynecol Oncol.

[33] Mangili G, Bergamini A, Taccagni G, Gentile C, Panina P, Vigano P (2012). Unraveling the two entities of endometrioid ovarian cancer: a single center clinical experience. Gynecol Oncol.

[34] Kim HS, Kim MA, Lee M, Suh DH, Kim K, No JH (2015). Effect of endometriosis on the prognosis of ovarian clear cell carcinoma: a two-center cohort study and meta-analysis. Ann Surg Oncol.

[35] Goff BA, Sainz de la Cuesta R, Muntz HG, Fleischhacker D, Ek M, Rice LW (1996). Clear cell carcinoma of the ovary: a distinct histologic type with poor prognosis and resistance to platinum-based chemotherapy in stage III disease. Gynecol Oncol.

[36] Mizuno M, Kikkawa F, Shibata K, Kajiyama H, Ino K, Kawai M (2006). Long-term follow-up and prognostic factor analysis in clear cell adenocarcinoma of the ovary. J Surg Oncol.

[37] Behbakht K, Randall TC, Benjamin I, Morgan MA, King S, Rubin SC (1998). Clinical characteristics of clear cell carcinoma of the ovary. Gynecol Oncol.

[38] Itamochi H, Kigawa J, Sugiyama T, Kikuchi Y, Suzuki M, Terakawa N (2002). Low proliferation activity may be associated with chemoresistance in clear cell carcinoma of the ovary. Obstet Gynecol.

[39] Itamochi H, Kigawa J, Terakawa N (2008). Mechanisms of chemoresistance and poor prognosis in ovarian clear cell carcinoma. Cancer Sci.

[40] Kawaguchi W, Itamochi H, Kigawa J, Kanamori Y, Oishi T, Shimada M (2007). Simultaneous inhibition of the mitogen-activated protein kinase kinase and phosphatidylinositol 3′-kinase pathways enhances sensitivity to paclitaxel in ovarian carcinoma. Cancer Sci.

[41] Fujimura M, Hidaka T, Saito S (2002). Selective inhibition of the epidermal growth factor receptor by ZD1839 decreases the growth and invasion of ovarian clear cell adenocarcinoma cells. Clin Cancer Res.

